# Acne: Its Etiology, Pathology and Treatment

**Published:** 1885-11

**Authors:** 


**Acne: Its Etiology, Pathology and Treatment.** A practical treatise based on the study of 1,500 cases of sebaceous diseases, by L. DUNCAN BULKLEY, A. M., M. D., Physician to the New York Skin and Cancer Hospital, Dermatologist at the New York Hospital, etc. New York and London: G. P. Putnam's Sons. 1885.

We have found all that Dr. Bulkley has written on subjects connected with dermatology to be not only worth reading, but worthy of study, and this last work of his is no exception. It is a comprehensive—indeed, an exhaustive—study of the diseases of the sebaceous glands; and when we consider that there are not less than 600,000 of these minute structures on each individual, it will appear that the proper performance of their functions has a relation not only to comeliness, but also to the general health. While we find in this volume a good deal of matter of purely scientific and literary interest, there is no curtailment of the practical side of the subject, and the practitioner will find full details of treatment, even to the minutiæ of diet and hygiene. It is a book for the general practitioner as well as the specialist, and we most heartily commend it.

